# Preparation and Identification of Antioxidative Peptides from Pacific Herring (*Clupea pallasii*) Protein

**DOI:** 10.3390/molecules24101946

**Published:** 2019-05-21

**Authors:** Xueqin Wang, Huahua Yu, Ronge Xing, Song Liu, Xiaolin Chen, Pengcheng Li

**Affiliations:** 1 Key Laboratory of Experimental Marine Biology, Center for Ocean Mega-Science, Institute of Oceanology, Chinese Academy of Sciences, No 7, Nanhai Road, Qingdao 266071, China; xueqinwang@qdio.ac.cn (X.W.); yuhuahua@qdio.ac.cn (H.Y.); xingronge@qdio.ac.cn (R.X.); sliu@qdio.ac.cn (S.L.); chenxl@qdio.ac.cn (X.C.); 2 Laboratory for Marine Drugs and Bioproducts of Qingdao National Laboratory for Marine Science and Technology, No 1, Wenhai Road, Qingdao 266237, China

**Keywords:** pacific herring, protein hydrolysate, peptides, antioxidant activity, purification

## Abstract

The aim of this study was to isolate and purify antioxidative peptides from Pacific herring (*Clupea pallasii*) protein. Five enzymes (pepsin, trypsin, papain, flavourzyme, and neutrase) were used for protein hydrolysis, and Pacific herring protein hydrolysates (PHPH) were separated by ultrafiltration. The fraction with the molecular weight below 3500 Da exhibited the highest in vitro antioxidant activities and cellular antioxidant activity. The PHPH was isolated and purified by consecutive chromatographic methods including gel filtration chromatography and reverse high-performance liquid chromatography (RP-HPLC). The purified antioxidant peptides were identified as Leu-His-Asp-Glu-Leu-Thr (MW = 726.35 Da) and Lys-Glu-Glu-Lys-Phe-Glu (MW = 808.40 Da), and the IC_50_ values of cellular antioxidant activity were 1.19 ± 0.05 mg/mL and 1.04 ± 0.06 mg/mL. The results demonstrate that is possible to produce natural antioxidative peptides from Pacific herring protein via enzymatic hydrolysis and purification.

## 1. Introduction

Antioxidant peptides, which contained residues 5–16 amino acid, are mainly produced through protein hydrolysis by enzyme. Antioxidative peptides from foods are considered to be safe and healthy compounds with low molecular weight, low cost, high activity, and easy absorption [1]. Reactive oxygen species (ROS) are generated by chemical reactions and metabolic processes, including tissue injury or DNA damage [2]. When the ROS or oxidants levels out of balance, it would cause numerous chronic diseases, including cancer, diabetes, et al. [3]. It has been reported that antioxidative peptides keep cells safe from damage by ROS through the induction of genes. Therefore, it is necessary to develop high antioxidant activity and safety antioxidant peptides from a nature food-based [4].

Antioxidant activity has been reported for protein hydrolysates prepared from various fish sources, such as capelin, mackerel, yellowfin sole, Alaska pollack, Atlantic salmon, hoki, conger eel, and scad [5]. Moreover, the researchers used many methods to measure the antioxidant activities of hydrolysates, and some researchers predicted the in vivo activity through the in vitro antioxidant activity assay, that has been questioned for a number of reasons [6]. As the best measures, animal and human models also have the disadvantage, including time-consuming, expensive and harsh experimental conditions [7]. Cell culture models provided an approach that is rapid and cost effective, and the model can reflect some phenomena, including distribution, regarding uptake and metabolism. Hepatocellular carcinoma (HepG2) cell line is a reliable model for evaluating the antioxidant activity of samples [8]. There are many studies have used HepG2 cells model to investigate the antioxidant effects of samples [9,10,11].

Pacific herring (*Clupea pallasii*) is distributed in the North Pacific and have critical ecological and commercial roles in oceanic and coastal waters [12]. Pacific herring is characterized by high content protein, population abundance, and low production cost. In recent decades, studies of Pacific herring, e.g., Hoyle and Merritt [13], have used different proteases to produce fish protein hydrolysates from whole Atlantic herring (*Clupea harengus*) and have investigated their characteristics; Sathivel et al. [14] studied the antioxidative properties of hydrolysed herring (*C. harengus*) and herring by-products. However, research on Pacific herring has been largely focused on resource quantity and distribution, and few studies of the preparation, purification and identification of Pacific herring have been conducted.

In this study, we used response surface method to optimize the hydrolytic condition of Pacific herring protein hydrolysate (PHPH), and the PHPH with molecular weights below 3500 Da were chosen as potential antioxidant peptide resources. Furthermore, the PHPH were separated using a series of chromatographic techniques and the amino acid sequences were identified, providing a theoretical basis for the high-value utilization of Pacific herring.

## 2. Results and Discussion

### 2.1. Cytotoxic Effects of PHPH on HepG2 Cells

**Figure 1:**
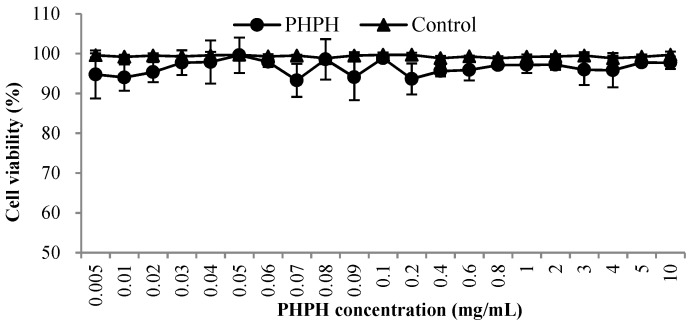
Toxic effects of PHPH on HepG2 cell viability. The PHPH was hydrolysed by trypsin.

An objective of this work was to investigate the effects of PHPH with different concentrations (0.005–10 mg/mL) on the cell viability of HepG2 cells in culture because high doses of PHPH can be toxic in cell culture systems [15]. As shown in Figure 1, the cell viability values were greater than 93% when the concentrations from 0.005 mg/mL to 10 mg/mL, the result was close to the control group and exhibited that PHPH was almost non-toxic to HepG2 cells. Finally, the concentrations with above 90% cell viability were selected for further analyses.

### 2.2. Selections of Proteolytic Enzymes 

**Figure 2:**
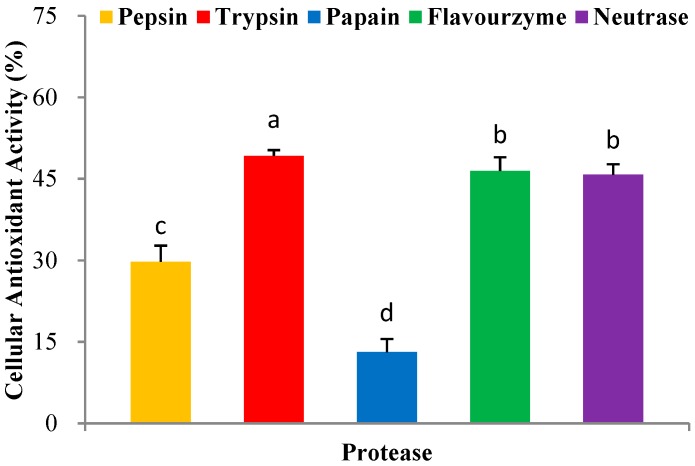
Cellular antioxidant activity of different enzymatic hydrolysis products. Bar graphs followed by different letters indicate significant differences (*p* < 0.05).

In recent years, the enzymatic hydrolysis of proteins of marine organisms has been extensively studied to identify bioactive peptides [16]. Here, we used five proteases including pepsin, trypsin, papain, flavourzyme, and neutrase, for the hydrolytic production of PHPH. The H_2_O_2_ was added in the HepG2 cells, and causing cells oxidative stress damage. The PHPH could clear hydroxyl radical from the H_2_O_2_ and protect the cells against oxidative damage. Then, we think the PHPH could protect the HepG2 cells via the effect of its antioxidative activity. In this section, the protease exhibited higher cellular antioxidant activity would be an optimum one for further research. As shown in Figure 2, PHPH (10 mg/mL) treated with trypsin exhibited the highest cellular antioxidant activity (49.21% ± 1.07%), followed by flavourzyme, neutrase, pepsin and papain. Therefore, trypsin was chosen as the optimum one for the next experiment. 

### 2.3. Optimization of PHPH by Response Surface Methodology (RSM) 

In this work, a single-factor experiment was conducted to investigate the effects of hydrolysis conditions on cellular antioxidant activity; based on single-factor experiment, RSM was used to obtain the optimum conditions for the preparation of PHPH. As shown in Table 1, X_1_, X_2_, X_3_, X_4_ and X_5_ represented enzyme concentration (U/g), extraction time (h), pH, water/material ratio (*v*/*w*) and extraction temperature (°C), respectively; and Y represented cellular antioxidant activity (%). The range of the cellular antioxidant activity of PHPH was from 25.73% to 43.22%. The data were analysed via multiple regression analysis using Design-Expert software, which yielded the following polynomial equation:

**Y** = +40.95 + 2.40X_1_ + 2.45X_2_ + 1.10X_3_ + 0.71X_4_ + 2.39X_5_ + 1.37X_1_X_2_ − 0.81X_1_X_3_ − 1.61X_1_X_4_ − 2.45X_1_X_5_ − 1.26X_2_X_3_ − 1.08X_2_X_4_ + 0.34X_2_X_5_ + 0.077X_3_X_4_ − 3.816 × 10^−3^X_3_X_5_ + 0.27X_4_X_5_ − 4.09X_1_^2^ − 0.11X_2_^2^ − 2.59X_3_^2^ − 1.47X_4_^2^ − 3.51X_5_^2^ (1)

Analysis of variance (ANOVA) results were presented in Table 2. F value of the model was 15.34, and P value was less than 0.0001, implying the model was significant and could be used for the optimization [17]. The variables with significant effects (*p* < 0.01) on the cellular antioxidant activity of PHPH were X_1_, X_2_, X_3_, X_5_, X_1_X_4_, X_1_X_5_, X_1_^2^, X_3_^2^, X_4_^2^, and X_5_^2^. The lack of fit value of 0.6059 indicated it was not significant relative to the pure error. The model showedgood fit with the experimental data, with high R^2^ (0.9246) and Adj. R^2^ (0.8643). These findings suggested that the hydrolysis of PHPH could be reliably analysed and predicted by the model.

A 3D response surface was a graphical representation of a regression equation [18], the response surface plots revealed effects of two factors on cellular antioxidant activity; the effects of the remaining factors were maintained at zero [19]. The response surface plots would suggest well-defined optimum conditions when their shapes were convex [20].

**Figure 3:**
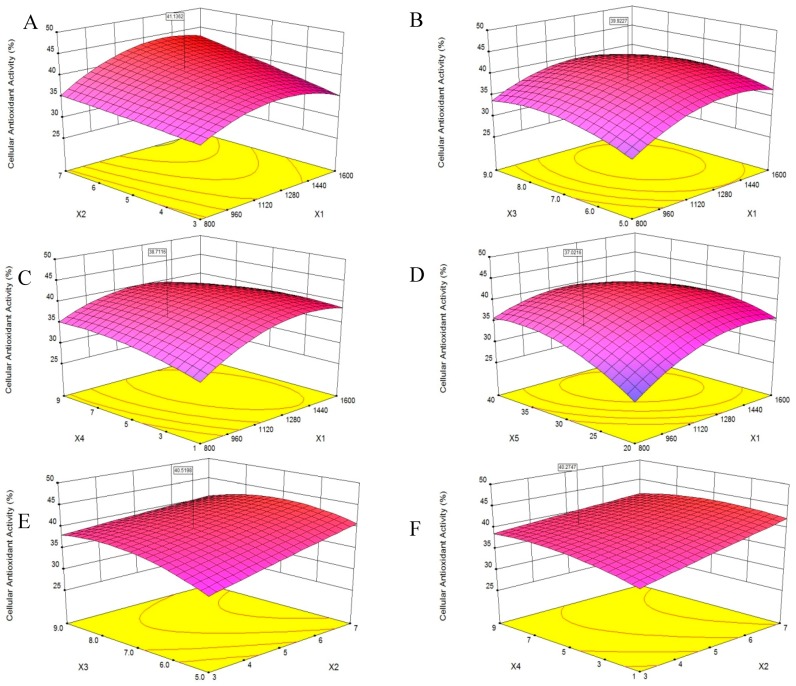

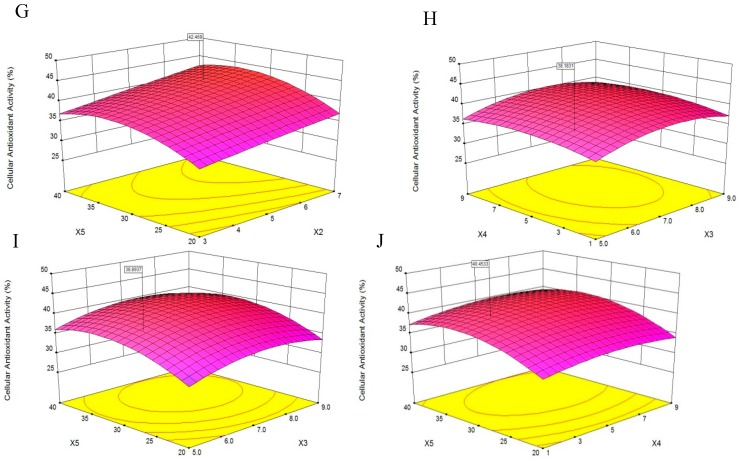
Response surface plots on the cellular antioxidant activity of PHPH. X_1_, X_2_, X_3_, X_4_ and X_5_ represent enzyme concentration, extraction time, pH, water/material ratio and extraction temperature. Figures (**A**–**J**) meant the response surface plots displayed the effects of two factors on cellular antioxidant activity.

The results presented in Figure 3A showed that cellular antioxidant activity increased as X_1_ increased from 800 U/g to 1300 U/g and decreased slightly as X_1_ increased from 1300 U/g to 1600 U/g, indicating that X_1_ was a critical factor in enzymatic hydrolysis. This finding was consistent with the report of Batista et al. [21] that an increase in X_1_ might result in a reduced rate of hydrolysis. Additionally, when the X_2_ increased from 3 h to 7 h, the cellular antioxidant activity increased slightly, and the same trend was shown in Figure 3E–G.

Figure 3B,E,H,I showed that the cellular antioxidant activity increasing slightly when X_3_ increased from 5.0 to 9.0, and then plateaued. Maximum cellular antioxidant activity was achieved when X_1_ and X_3_ were 1450 U/g and 7.0, respectively.

According to Figure 3C, as the X_4_ increased from 1 to 9, the cellular antioxidant activity increased slightly, and the same trend was shown in Figure 3F. Additionally, the cellular antioxidant activity initially increased and then decreased slightly as the X_4_ increased from 1 to 9, as shown in Figure 3H,J. We speculated that a higher X_4_ may dilute the X_1_ and slow the rate of enzyme reactions [22].

Figure 3D, showed that as the X_5_ increased from 20 °C to 40 °C, the cellular antioxidant activity increased significantly. Feng et al. [19] reported that increases in temperature helped disperse the solutes and increase yield. Maximum cellular antioxidant activity was achieved when X_1_ and X_5_ were 1400 U/g and 32 °C, respectively.

The optimum level of cellular antioxidant activity, 44.56%, was achieved at X_1_ of 1394.02 U/g, X_2_ of 7.0 h, X_3_ of 6.78, X_4_ of 3.51 *v*/*w* and X_5_ of 32.06 °C. The experimental cellular antioxidant activity value agreed with the value predicted by the model within a 95% confidence interval. This model was suitable for the estimation of experimental values and useful for the optimization predictions of the extraction. The extraction yield of PHPH under this condition was 18.49 g/(100 g original herring protein).

### 2.4. Amino Acid Composition of PHPH

The antioxidative activity of a protein hydrolysate fraction could be affected by the amino acid composition [23]. 

As shown in Table 3, the PHPH was rich in Glu, Asp, Leu, Lys, and Arg. Some studies have reported that Arg, Glu, Leu, and Asp can enhance the antioxidant activity of peptides [24,25]. Additionally, the ratios of ∑EAA/∑NEAA and ∑EAA/∑AA in PHPH were 0.70 and 0.41, respectively; and the FAO/WHO recommended values were 0.6 and 0.4, respectively. The results exhibited that the PHPH contained abundant amino acids and could be the desirable antioxidative peptides. 

### 2.5. Purification of PHPH

#### 2.5.1. Fractions of PHPH by Ultrafiltration 

**Figure 4:**
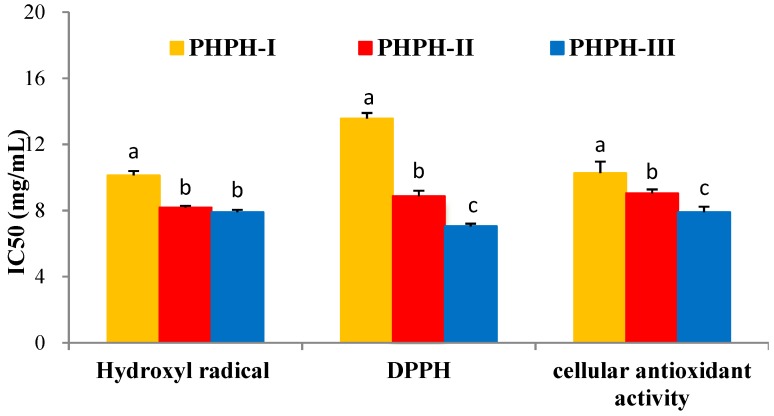
Antioxidant activities of different fractions from PHPH. Bar graphs followed by different letters indicated significant differences (*p* < 0.05).

PHPH was fractioned by ultrafiltration with two MWCO membranes (10,000 Da and 3500 Da). Three fractions were separated and named PHPH-I (MW > 10,000 Da), PHPH-II (MW = 3500−10,000 Da) and PHPH-III (MW < 3500 Da). The yield of PHPH-III was 85.46g/(100 g PHPH). Half maximal inhibitory concentration (IC_50_) values of these fractions were given in Figure 4, PHPH-III showed the highest hydroxyl radical scavenging activity, with IC_50_ value of 7.91 ± 0.13 mg/mL. The 1,1-dipheny-2-picryhydrazyl (DPPH) radical scavenging activity of PHPH-I, PHPH-II and PHPH-III were 13.56 ± 0.34, 8.87 ± 0.33 and 7.05 ± 0.15 mg/mL, respectively. Furthermore, the IC_50_ value of cellular antioxidant activity in PHPH-III was 7.90 ± 0.33 mg/mL, which was significantly higher than the activities in PHPH-I and PHPH-II of 10.26 ± 0.69 and 9.04 ± 0.23 mg/mL, respectively.

These results were consistent with Wang et al., who reported that peptides with molecular weights below 3000 Da derived from blue mussel (*Mytilus edulis*) protein hydrolysate exhibited higher antioxidant activity than did those with higher molecular weights [26]. It has been reported that the molecular weight distribution can affect the antioxidant activity of hydrolysates, and the peptides with lower molecular weights can cross the intestinal barrier more easily than those with higher weights to exert their biological effects [27].

#### 2.5.2. Gel Filtration Chromatography of PHPH-III

**Figure 5:**
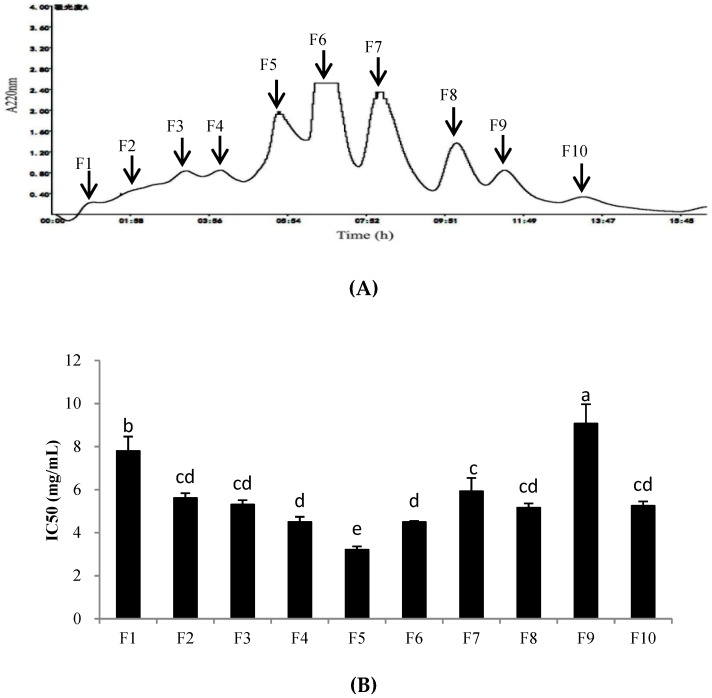
Separation chromatogram for the PHPH-III by a Sephadex G-25 gel filtration column (**A**) and cellular antioxidant activity of the eluted peak (**B**). Bar graphs followed by different letters indicated significant differences (*p* < 0.05). Fractions F1–F10 were separated from Sephadex G-25 gel filtration column.

PHPH-III, which was obtained by treatment with ultrafiltration and displayed the highest cellular antioxidant activity among the fractions, was further fractioned by a Sephadex G-25 gel filtration column. The fractions were separated and designated F1–F10 (Figure 5A). These fractions were collected, freeze-dried, and determined the cellular antioxidant activities. As shown in Figure 5B, fraction F5 exhibited the highest level of cellular antioxidant activity with the IC_50_ value of 3.21 ± 0.15 mg/mL. The yield of PHPH-III-5 was 20.16 mg/(250 mg PHPH-III).

#### 2.5.3. RP-HPLC Analysis

**Figure 6:**
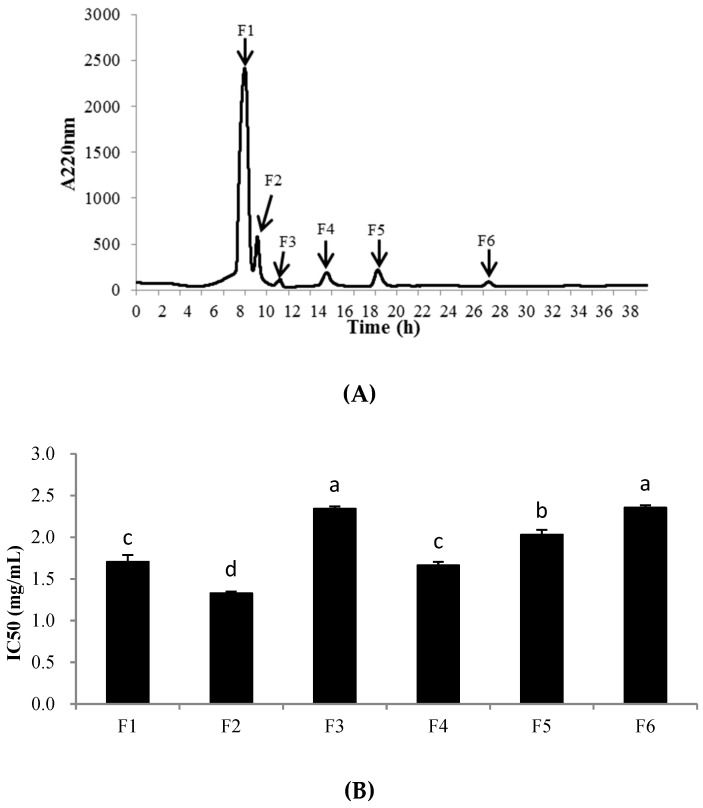
Separation chromatogram for the PHPH-III-5 by an XBridge® BEH C18 column (**A**) and cellular antioxidant activity of the eluted peaks (**B**). Bar graphs followed by different letters indicated significant differences (*p* < 0.05). Fractions F1–F6 were separated from XBridge® BEH C18 column.

An XBridge® BEH C18 column was used for further purification of the fraction PHPH-III-5. As shown in Figure 6, 6 peaks, designated F1-F6, were collected separately. Each fraction was freeze-dried and determined the cellular antioxidant activities. The result showed that F2 exhibited the highest cellular antioxidant activity with the IC_50_ value of 1.33 ± 0.02 mg/mL (Figure 6B); this activity was significantly higher than the activities of the other fractions at the same concentration (*p* < 0.05). The yield of PHPH-III-5-2 was 2.56 mg/(20 mg PHPH-III-5).

### 2.6. Characterization of PHPH

**Figure 7:**
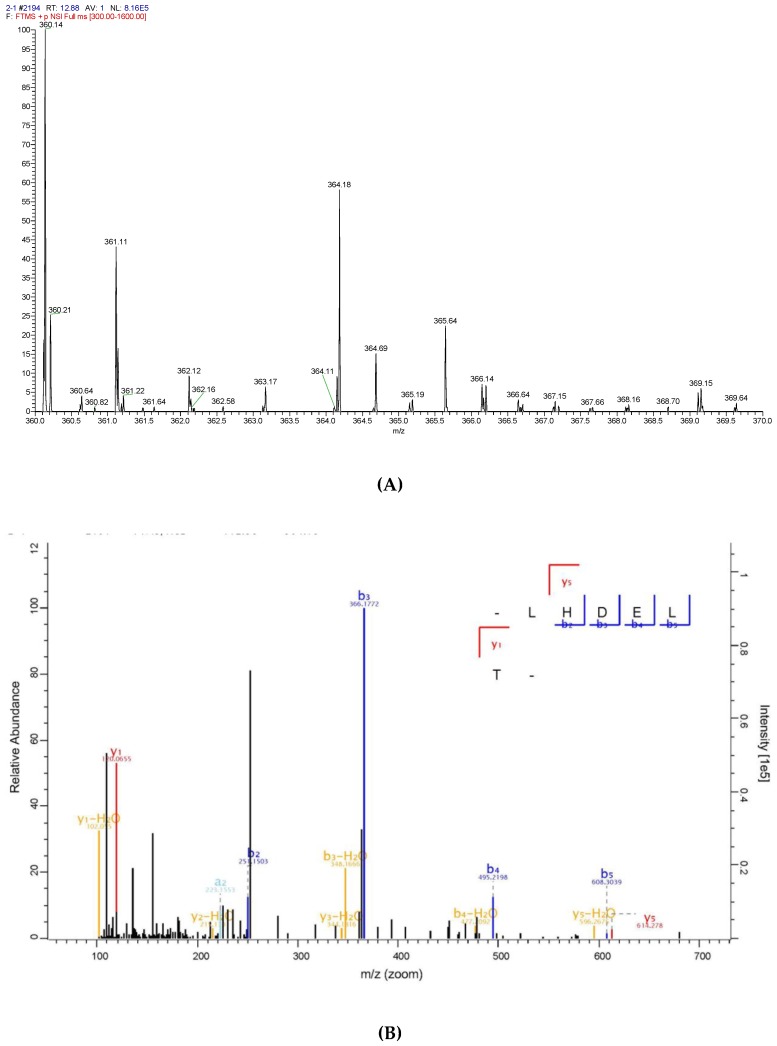
Identification of the peptide P1 from PHPH-III-5-2. Mass spectrum of the chromatographic P1 (**A**), and the collision induced fragmentation of P1 (**B**). The sequence of P1 was displayed with the fragment ions observed in the MS/MS spectrum.

**Figure 8:**
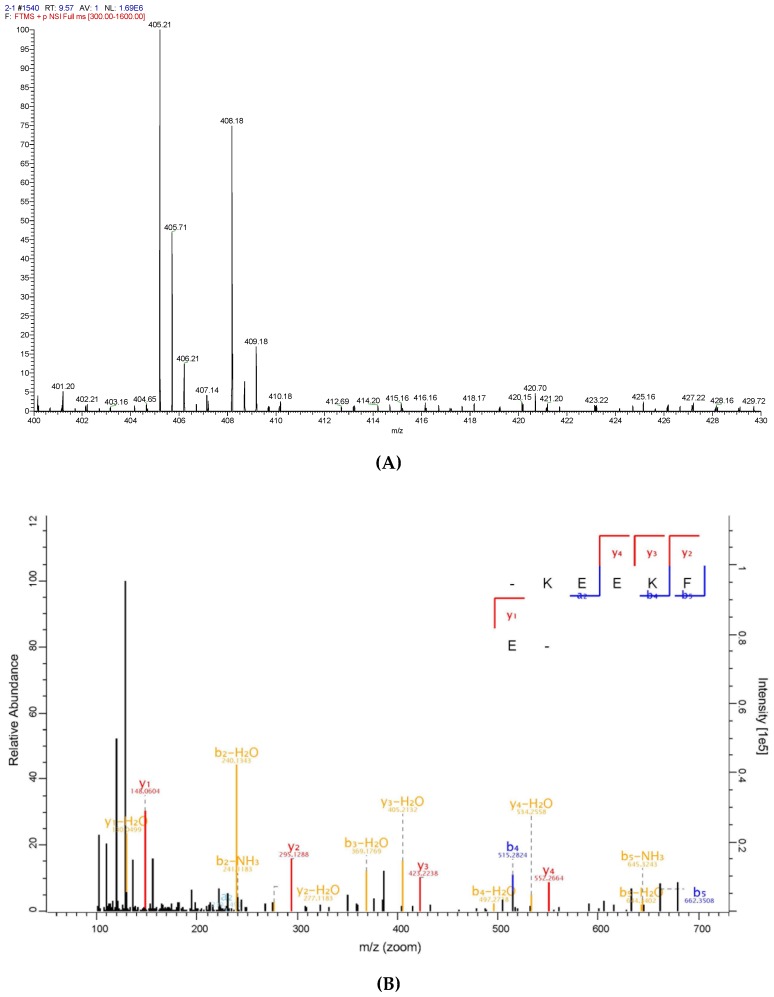
Identification of the peptide P2 from PHPH-III-5-2. Mass spectrum of the chromatographic P1 (**A**), and the collision induced fragmentation of P2 (**B**). The sequence of P2 was displayed with the fragment ions obse.rved in the MS/MS spectrum.

To identify the antioxidative peptide, the mass spectrometer used to analyse PHPH-III-5-2. As shown in Figure 7 and Figure 8, two high-score peptides, P1 and P2 were obtained. The MS/MS spectrum of two charged ions with *m*/*z* at 364.18 Da and 405.21 Da were shown in Figure 7B and Figure 8B, respectively, and the molecular weight of peptides P1 and P2 were determined to be 726.35 Da and 808.40 Da, respectively. Additionally, the amino acid sequence of peptide P1 was identified as Leu-His-Asp-Glu-Leu-Thr and peptide P2 was identified as Lys-Glu-Glu-Lys-Phe-Glu. 

Additionally, to evaluate the antioxidant activities of P1 and P2, the cellular antioxidant activity and radical scavenging activities were investigated. As shown in Table 4, P1 exhibited the hydroxyl radical scavenging activity, DPPH radical scavenging activity, and cellular antioxidant activity with the IC_50_ values of 4.57 ± 0.24 mg/mL, 5.14 ± 0.32 mg/mL, and 1.19 ± 0.05 mg/mL, respectively; P2 exhibited higher antioxidant activities than P1, the IC_50_ values were 3.78 ± 0.17 mg/mL, 4.37 ± 0.26 mg/mL, and 1.04 ± 0.06 mg/mL, respectively. Research findings indicated that peptides with 2–6 amino acids were absorbed more readily in comparison to protein and free amino acids [1]. Yang et al. [28] had identified the protein hydrolysate of Hairtail (*Trichiurus japonicas*) muscle, and the EC_50_ values of DPPH scavenging activities were 0.626 mg/mL to 0.902 mg/mL; Najafian et al. [29] had studied the peptide from fermented fish (*pekasam*) and the identified peptides exhibited the IC_50_ values of DPPH scavenging activities were0.897 mg/mL and 1.38 mg/mL, respectively.

Many studies have reported that the antioxidative activity of peptides can be affected by the amino acid sequence [30]. For example, Leu could enhance the scavenging activities of peptides [31,32]; Kim et al. and Rajapakse et al. [33,34] studied the activities of individual amino acids and reported that Glu, Lys and Phe consistently showed higher antioxidant activity than the other amino acids. Furthermore, peptides P1 and P2 contained the amino acids related to antioxidant activity, which was consistent with those reported from other studies from fish sources [35,36,37].

## 3. Materials and Methods 

### 3.1. Materials and Chemicals

Individuals of Pacific herring (*Clupea pallasii*) were purchased from a seafood market in Qingdao, China. Whole fish were transported on ice to the laboratory. Upon arrival, the fish were washed, and the fish meat was collected, minced, and freezed. Five proteases (papain, flavourzyme, pepsin, trypsin, and neutrase) were obtained from Solarbio Co. (Beijing, China). An ultrafiltration (UF) system and UF membranes were buy from Laungy Co., Ltd. (Shanghai, China). Hepatocellular carcinoma (HepG2) cells were obtained from Qingdao University (Shandong, China). Trifluoroacetic acid (TFA), acetonitrile, formic acid and methanol were of guaranteed reagent and purchased from Sigma Chemical Co. (St Louis, MO, USA). All other chemicals and solvents were of analytical reagent, and purchased from Sinopharm Chemical Reagent Co., Ltd (Shanghai, China).

### 3.2. Preparation of PHPH

Five proteases were studied to determine the optimal enzyme. The Pacific herring protein was mixed with deionized water at a ratio of 1:10 *v*/*w*. The mixtures were adjusted to the required pH with 0.01 mol/L NaOH or HCl and heated in a water bath to the required temperature before proteases were added in a proper proportion based on enzyme activity. After reaction finished, the mixtures were heated for 15 min at 100 °C in order to end up hydrolysis followed by centrifugation at 18,000× *g* for 10 min and the supernatants were concentrated and freeze-dried. The cellular antioxidant activities of five enzymatic hydrolysis products with the concentration of 10 mg/mL were determined.

The protease, which exhibited the highest cellular antioxidant activity, would be chosen for the next experiment. Additionally, we used RSM to obtain the optimum hydrolysis conditions [38]. 

### 3.3. Optimization of PHPH Preparative Conditions 

It was important to choose suitable variables with appropriate levels before conducting the RSM experiment [39]. Generally speaking, there were five enzymatic hydrolysis reaction variables, such as extraction temperature, enzyme concentration, extraction time, pH and water/material ratio. Firstly, we used single-factor experiment to confirm the appropriate level, and then used the Box-Behnken design (BBD) to design the experiment, each variable contained five levels, and the designed experiment had 46 runs.

### 3.4. Amino Acid Analysis

The amino acid analysis method was in accordance with Luo et al. [40]. The PHPH was hydrolysated with 6 mol/L HCl at 110 °C for 24 h, and constant volume to 50 mL. Then, 1 ml sample suck to evaporate, then dissolved with water and evaporated, the residue was diluted with 1 mL of buffer (pH 2.2) and detected with a S433D amino acid analyser (SYKAM, Eresing, Munich, Germany). This method could detect sixteen amino acids without tryptophan because of acid hydrolysis oxidise. 

### 3.5. Antioxidant Analysis of HepG2 Cells 

#### 3.5.1. Cytotoxic Effects of PHPH on HepG2 Cells

HepG2 cells are always used for evaluating the antioxidant activity of samples [41]. We used methyl thiazolyl tetrazolium (MTT) assay to measure the inhibition of HepG2 [10]. The cells were cultivated in 96-well culture plates at the optimal temperature and humidity for 24 h. Next, the PHPH with different concentrations (100 µL) were added in the cells for another 24 h, and each well was added 20 µL of MTT for 3 h. Then, 150 µL dimethyl sulfoxide (DMSO) was added into the well and shaken for 10 min. Finally, we used a microplate reader (Bio-Rad, California, USA) to obtain the absorbance at a wavelength of 490 nm. The experiment was repeated 4 times. 

#### 3.5.2. Cellular Antioxidant Activity Determination

The cells were cultivated in 96-well culture plates at the optimal temperature and humidity for 24 h. Firstly, the PHPH (100 µL) with different concentrations were added in the cells for another 24 h, and then 1000 µM H_2_O_2_ (100 µL) was added for another 24 h. The MTT assay as described above was used to measure the viability of HepG2 to evaluate the cellular antioxidant activity of PHPH, in this section, the cellular antioxidant activity was defined as cell viability: 

Cell viability (%) = (A_1_ − A_0_)/(A_2_ − A_0_) × 100, where A_1_ is the absorbance with PHPH and H_2_O_2_, A_2_ is the absorbance without PHPH and H_2_O_2_, and A_0_ is the absorbance with H_2_O_2_. The experiment was repeated 4 times. 

### 3.6. Hydroxyl Radical Scavenging Activity

We measured the hydroxyl radicals scavenging activity of PHPH followed the description of Smeriglio et al. [42]. Various concentrations of the PHPH were mixed with 0.5 mL of EDTA-FeSO_4_ (2.0 mmol/L), 1.0 mL of phosphate buffer (PBS, pH 7.4), 1.0 mL of safranine T, and 1.0 mL of H_2_O_2_. The control group was added PBS replaced H_2_O_2_. After mixing and incubation for 60 min in 37 °C, absorbance was measured spectrophotometrically at 520 nm. The hydroxyl radicals scavenging activity was calculated according to the following equation,

Hydroxyl radical scavenging activity (%) = (A_sample_ − A_blank_)/(A_control_ − A_blank_) × 100. Half maximal inhibitory concentration of hydroxyl radical was used to calculate the antioxidant activity of sample.

### 3.7. DPPH Radical Scavenging Activity 

The DPPH radical assay is a simple and rapid method to test the scavenging activity of sample. We measured the DPPH radicals scavenging activity of PHPH followed the description of Blois [43]. 1.0 mL of DPPH solution (0.1 mM, dissolved in ethanol) was mixed with 3.0 mL of PHPH or distilled water in a test tube, after mixing and incubation for 30 min, the absorbance was determined at 517 nm. The blank group containing distilled water and ethanol. The DPPH radical scavenging activity was calculated according to the following equation,

DPPH radical scavenging activity (%) = (A_control_ − A_sample_)/(A_control_ − A_blank_) × 100. Half maximal inhibitory concentration of DPPH radical was used to calculate the antioxidant activity of sample.

### 3.8. IC_50_ Values Determination

IC_50_ values, defined as the concentration of PHPH (mg/mL) required inhibiting 50% of cellular antioxidant activity or radical scavenging activity, were determined by nonlinear regression from a plot of analysis of cellular antioxidant activity or radical scavenging activity versus the PHPH concentrations (SPSS 16.0 statistic software).

### 3.9. Purification of PHPH

#### 3.9.1. Ultrafiltration 

UF is often used to separate hydrolysates to obtain the target active fractions [44]. The PHPH was fractionated through UF membranes have a range of molecular weight cut-offs (MWCOs) of 10,000 Da and 3500 Da, respectively. Fractionates were designed as follows: PHPH-I (>10,000 Da), PHPH-II (3500–10,000 Da) and PHPH-III (<3500 Da). All fractions were frozen drying and refrigerated for further experiment.

#### 3.9.2. Gel Filtration Chromatography

PHPH-III (250mg/mL) was loaded onto a Sephadex G-25 gel filtration column (2.6 × 80 cm, GE Healthcare, Uppsala, Sweden), equilibrated with distilled water, and eluted with distilled water at a flow rate of 0.5 mL/min, each fraction monitored at 220 nm using an ultraviolet detector, and the freeze-dried fractions was determined using the cellular antioxidant activity assay. 

#### 3.9.3. Reverse-Phase High-Performance Liquid Chromatography (RP-HPLC)

The active fraction PHPH-III-5 was further loaded onto an XBridge® BEH C18 column (19 × 250 mm, Waters, USA) equilibrated with 0.1% (*v*/*v*) TFA and eluted with a linear gradient of acetonitrile (0–20% in 30 min) containing 0.1% TFA. The elution rate was 5.0 mL/min and each fraction monitored at 220 nm using the HPLC system and the freeze-dried fractions was determined using the cellular antioxidant activity assay. 

#### 3.9.4. Identification of Peptides by Mass Spectrometry

The fraction of PHPH-III-5-2 was loaded onto a C18 column eluted with a linear gradient of acetonitrile (0–90% in 40 min) containing 0.1% formic acid. Accurate molecular mass and amino acid sequence of PHPH-III-5-2 was determined by a Thermo Scientific Q Exactive mass spectrometer. Spectra were recorded over the mass/charge range 350–1800 (*m*/*z*).

### 3.10. Statistical Analysis

Data are presented as a means mean ± standard error of the mean (n = 3 or 4). Data were analysed using one-way analysis of variance (ANOVA). *p* < 0.05 indicated statistical significance. 

## 4. Conclusions

In this study, we optimized the enzymatic conditions for trypsin in Pacific herring (*C. pallasii*) protein, and the optimum hydrolysis conditions were an enzyme concentration of 1394.02 U/g, an extraction time of 7.0 h, a pH of 6.78, a water/material ratio of 3.51 *v*/*w*, and an extraction temperature of 32.06 °C. The purification and characterization of PHPH were reported for the first time in Pacific herring (*C. pallasii*) protein study, and the peptides Leu-His-Asp-Glu-Leu-Thr (MW = 726.35 Da) and Lys-Glu-Glu-Lys-Phe-Glu (MW = 808.40 Da) were identified. This research might contribute to a rational application of antioxidant peptides to explore functional foods and drugs to treat diseases associated with oxidative stress. Further research should be performed to investigate the in vivo effects of these peptides.

## Figures and Tables

**Table 1:** Experimental design and result of response surface.

Run Numbers	X_1_	X_2_	X_3_	X_4_	X_5_	Y
1	800	3	7	5	30	33.10
2	1600	3	7	5	30	34.82
3	800	7	7	5	30	36.03
4	1600	7	7	5	30	43.22
5	1200	5	5	1	30	36.64
6	1200	5	9	1	30	38.06
7	1200	5	5	9	30	36.34
8	1200	5	9	9	30	37.15
9	1200	3	7	5	20	32.29
10	1200	7	7	5	20	36.03
11	1200	3	7	5	40	37.60
12	1200	7	7	5	40	42.72
13	800	5	5	5	30	29.23
14	1600	5	5	5	30	36.81
15	800	5	9	5	30	33.86
16	1600	5	9	5	30	38.19
17	1200	5	7	1	20	34.06
18	1200	5	7	9	20	35.43
19	1200	5	7	1	40	35.63
20	1200	5	7	9	40	35.73
21	1200	3	5	5	30	32.55
22	1200	7	5	5	30	37.85
23	1200	3	9	5	30	37.93
24	1200	7	9	5	30	40.54
25	800	5	7	1	30	31.42
26	1600	5	7	1	30	34.81
27	800	5	7	9	30	31.34
28	1600	5	7	9	30	34.55
29	1200	5	5	5	20	31.51
30	1200	5	9	5	20	32.90
31	1200	5	5	5	40	33.42
32	1200	5	9	5	40	37.82
33	800	5	7	5	20	25.73
34	1600	5	7	5	20	30.13
35	800	5	7	5	40	36.72
36	1600	5	7	5	40	40.38
37	1200	3	7	1	30	34.52
38	1200	7	7	1	30	41.03
39	1200	3	7	9	30	42.03
40	1200	7	7	9	30	43.22
41	1200	5	7	5	30	39.07
42	1200	5	7	5	30	38.99
43	1200	5	7	5	30	42.80
44	1200	5	7	5	30	39.49
45	1200	5	7	5	30	42.14
46	1200	5	7	5	30	41.14

**Table 2:** ANOVA for the response of cellular antioxidant activity in the PHPH.

Variables	Sum of Squares	DF	Mean Square	F Value	P Value
Model	601.75	20	30.09	15.34	<0.0001
Residual	49.05	25	1.96		
Lack of fit	38.51	20	1.93	0.91	0.6059
Pure error	10.53	5	2.11		
Cor total	650.80	45			
R^2^	0.9246				
Adj. R^2^	0.8643				
Pred. R^2^	0.7400				
Adeq precision	17.822				
CV%	3.80				

**Table 3:** Amino acid composition of PHPH.

Amino Acid	Content (%)
Asp	4.93
Thr *	2.26
Ser	2.19
Glu	6.22
Gly	3.5
Ala	2.78
Gys	0.11
Val *	2.95
Met *	1.14
Ile *	2.45
Leu *	4.43
Tyr	1.07
Phe *	2.43
Lys *	4.3
His	1.35
Arg	4.01
Pro	2.37
∑AA	48.49
∑EAA	19.96
∑EAA/∑NEAA	0.70
∑EAA/∑AA	0.41

* expressed essential amino acids; ∑AA expressed total amino acid content; ∑EAA expressed essential amino acid content; ∑NEAA expressed non-essential amino acid content.

**Table 4:** IC_50_ values of P1 and P2.

Sample	IC_50_ (mg/mL)		
	Hydroxyl Radical Scavenging Activity	DPPH Radical Scavenging Activity	Cellular Antioxidant Activity
P1	4.57 ± 0.24	5.14 ± 0.32	1.19 ± 0.05
P2	3.78 ± 0.17	4.37 ± 0.26	1.04 ± 0.06
